# Tianfoshen oral liquid: a CFDA approved clinical traditional Chinese medicine, normalizes major cellular pathways disordered during colorectal carcinogenesis

**DOI:** 10.18632/oncotarget.14675

**Published:** 2017-01-16

**Authors:** Siliang Wang, Hengbin Wang, Yin Lu

**Affiliations:** ^1^ Jiangsu Key Laboratory for Pharmacology and Safety Evaluation of Chinese Materia Medica, School of Pharmacy, Nanjing University of Chinese Medicine, Nanjing, 210023, P. R. China; ^2^ Jiangsu Collaborative Innovation Center of Traditional Chinese Medicine (TCM) Prevention and Treatment of Tumor, Nanjing University of Chinese Medicine, Nanjing, 210023, P. R. China; ^3^ Changshu Leiyunshang Pharmaceutical Co., Ltd., Changshu, 215500, P. R. China

**Keywords:** Tianfoshen oral liquid, colorectal cancer, cell cycle arrest, apoptosis, angiogenesis

## Abstract

Colorectal cancer remains the third leading cause of cancer death worldwide, suggesting exploration of novel therapeutic avenues may be useful. In this study, therefore, we determined whether Tianfoshen oral liquid, a Chinese traditional medicine that has been used to treat non-small cell lung cancer, would be therapeutically beneficial for colorectal cancer patients. Our data show that Tianfoshen oral liquid effectively inhibits growth of colorectal cancer cells both *in vitro* and *in vivo*. We further employed a comprehensive strategy that included chemoinformatics, bioinformatics and network biology methods to unravel novel insights into the active compounds of Tianfoshen oral liquid and to identify the common therapeutic targets and processes for colorectal cancer treatment. We identified 276 major candidate targets for Tianfoshen oral liquid that are central to colorectal cancer progression. Gene enrichment analysis showed that these targets were associated with cell cycle, apoptosis, cancer-related angiogenesis, and chronic inflammation and related signaling pathways. We also validated experimentally the inhibitory effects of Tianfoshen oral liquid on these pathological processes, both *in vitro* and *in vivo*. In addition, we demonstrated that Tianfoshen oral liquid suppressed multiple relevant key players that sustain and promote colorectal cancer, which is suggests the potential therapeutic efficacy of Tianfoshen oral liquid in future colorectal cancer treatments.

## INTRODUCTION

Colorectal cancer (CRC) is a malignant cancer of the gastrointestinal tract with low overall survival rates [1, 2]. Inspite of tremendous progress in anti-cancer research, CRC patients experience high rates of mortality due to lack of early diagnostic techniques as well as clinical side effects plaguing current therapy [1]. The etiology of CRC is complex and influenced by factors such as smoking, drinking, obesity, high-fat low-fiber diet, inflammatory bowel disease and family medical history [3–6]. The traditional Chinese medicine (TCM) has been extensively used as an alternate treatment for cancers and Tianfoshen oral liquid (TFS) that is composed of eight medicinal herbs has been one such formulation [7, 8]. TFS has been widely produced in China according to the standard of quality control in the Chinese Pharmacopoeia and used to clinically treat non-small cell lung carcinoma (NSCLC) [74]. The TFS formula induces apoptosis in NSCLC cells, inhibiting their migration and invasion and thereby extending life span and improving the quality of life of cancer patients [74]. The lung and the large intestine are interior-exteriorly related [73], which is one of the most important content of the basic theory of TCM, promote us cannot help to speculating whether this formula can be used as an effective therapeutic for colorectal cancer. Our previous studies demonstrated that TFS could significantly inhibit human CRC growth *in vitro* and *in vivo*. However, mechanisms underlying the ant-cancer effects of TFS remained largely unknown and had to be further investigated.

Generally, the traditional Chinese medicines exert therapeutic effects on multiple targets and pathways of human body through their complex active components which cannot be accurately detected solely by conventional methods [9, 10]. Therefore, we employed a comprehensive approach that combined prediction of active compounds based on a range of pharmacokinetic parameters and excavate the multiple drug targets by network analysis based on the existing databases. This strategy was previously used to clarify the synergistic effects and mechanisms of multi-component, multi-target agents including other TCM formulas [11–13]. In addition, we conducted a wide range of experiments to further elucidate the mechanisms by which TFS exerted its therapeutic effects on CRC.

## RESULTS

### TFS inhibited CRC cell growth *in vitro* and *in vivo*

**Figure 1:**
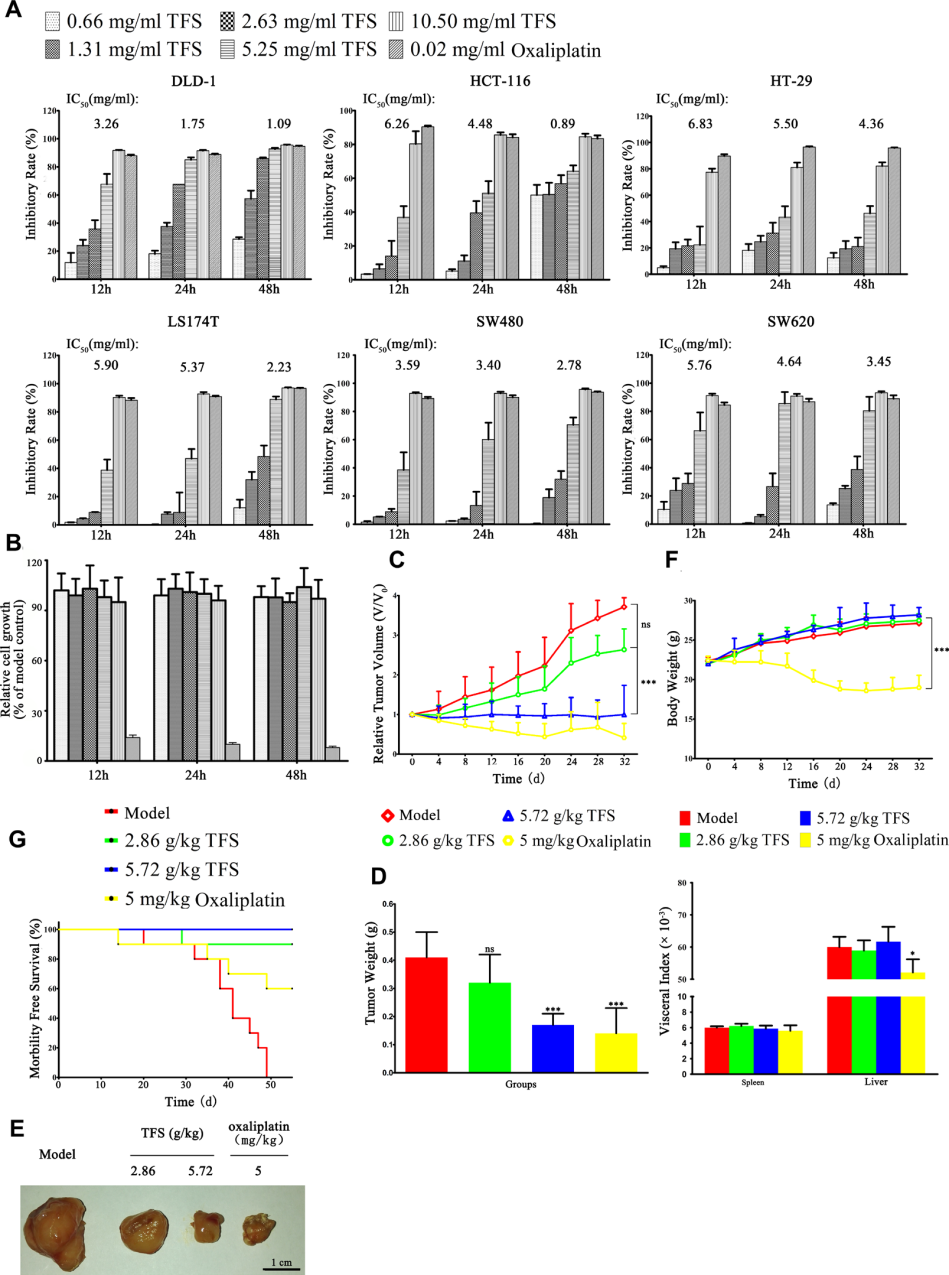
*In vitro* and *In vivo* effects of TFS on growth of CRC cell lines (**A**) Multiple CRC cell lines (SW480, SW620, HT-29, HCT-116, DLD-1 and LS174T) were treated with indicated concentrations of TFS (0.66, 1.31, 2.63, 5.25, 10.5 mg/ml) or oxaliplatin (0.02 mg/ml) for 12 h, 24 h and 48 h, respectively. The cell growth parameters were documented and calculated by the IncuCyte ZOOM^®^ live cell imaging system in comparison to the control group (saline treated). The data are presented as mean ± SD. The concentration of TFS resulting in 50% inhibition of control growth (IC_50_) was calculated by the SPSS statistics software using Probit model. (**B**) Colonic epithelial cells NCM460 were treated with indicated concentrations of TFS and oxaliplatin for 12 h, 24 h and 48 h, respectively. The cell growth parameters were analyzed as above. The data are presented as mean ± SD. (**C**) Tumor volume changes of mice treated with TFS (2.86 g/kg and 5.72 g/kg), oxaliplatin (5 mg/kg) and normal saline (model), respectively, are shown. Data are presented as mean ± SD (*n* = 10). ^**^*P < 0.001 (versus model). (**D**) Weight of tumors collected from different treatment groups of mice on day 32 is shown. Data are presented as mean ± SD (*n* = 10). ^**^*P < 0.001 (versus model). (**E**) Photographs of representative tumor blocks collected from different treatment groups of mice on day 32 are shown. (**F**) Body weight (above) and visceral index (below) changes of mice in different treatment groups on day 32 are shown. Data are presented as mean ± SD (*n* = 10). *P < 0.05, ^**^*P < 0.001 (versus model). (**G**) Survival rates of mice in different treatment groups at 55 d are shown.

Since TFS has been successfully used to clinically treat NSCLC, we investigated the *in vitro* effects of TFS on the growth of multiple human CRC cells (SW480, SW620, HT-29, HCT-116, DLD-1 and LS174T). TFS inhibited CRC growth in a dose and time dependent manner (Figure 1A; representative time-lapse movie in Supplementary Videos 1–10 ). Analysis of the IC_50_ values from each cancer cell line showed that TFS exerted 50% inhibition under 6 mg/ml after 48 h. On the other hand, TFS had little effect on the colon epithelial cells, NCM460 when subjected to a similar treatment analysis (Figure 1B). These results revealed the specific inhibitory effects of TFS on cancer cells.

To verify the effect of TFS on the growth of human CRC *in vivo*, nude mice bearing subcutaneous HT-29 xenografts that were treated with either 2.86 g/kg or 5.72 g/kg TFS for 5 weeks were compared with control mice that received either normal saline or oxaliplatin (5 mg/kg), respectively. The results showed that TFS effectively inhibited tumor progression (Figure 1C–1G). We observed that the relative tumor volume at day 32 was 2.63, 0.99, 0.41 and 3.71 for mice treated with TFS (2.86 g/kg), TFS (5.72 g/kg), oxaliplatin and normal saline, respectively (Figure 1C); The average tumor weight at day 32 of for the TFS-treated groups were 0.32 g and 0.17 g, respectively in comparison to 0.41g for the normal saline group and 0.12 g for the oxaliplatin group (Figure 1D). We further photographed the isolated day 32 tumor blocks to demonstrate that TFS treatment inhibited the growth of the xenografted HT-29 tumor compared with the normal saline control (Figure 1E). In addition, TFS did not cause any adverse side effects as visualized by weight and visceral index of mice (Figure 1F). The Kaplan–Meier survival analysis showed that all the mice treated with TFS survived the 55 day experimental period whereas all the control group mice died within 50 days (Figure 1G). Analysis of the therapeutic performance of TFS with SW480 cell-bearing nude mice demonstrated similar results (Supplementary Figure 1). Therefore, our experiments clearly demonstrated that TFS inhibited the growth of human CRC both *in vitro* and *in vivo* in a dose-dependent manner.

### Potential pharmacological mechanisms of TFS

### Candidate compound screening for TFS

TFS is composed of eight medicinal herbs, including, *Radix Panacis Quinquefolii* (RPQ), *Venenum Bufonis* (VB), *Radix Asparagi* (RA), *Bulbus* *Iphigenia Indica* (BII), *Radix Acanthopanax obouatus* (RAO), *Radix Actinidia chinensis Planch*. (RACP), *Fructus Hippophae* (FH) and *Fructus Citri Sarcodactylis* (FCS). Therefore, we combined oral bioavailability (OB) screening with drug-likeness evaluation to identify the active compounds in TFS [14]. We harvested 86 potential compounds with appropriate values for these two parameters from the herbal constituents of TFS. Further, 50 compounds with lower OB or drug-likeness index that exhibited extensive pharmacological activities and were typical components of herbal drugs were also collected as the candidate active compounds. The 136 compounds from the eight herbs that were considered as “candidate compounds” are listed in Supplementary Table 1. The eight different herbs, RPQ, VB, RA, BII, RAO, RACP, FH and FCS contributed 25, 23, 9, 16, 7, 14, 52 and 9 candidate compounds, respectively. Among the 136 candidate compounds, ten were widely distributed in the multiple herbs of TFS and had been certified to demonstrate diverse biological effects. For example, β-sitosterol that was present in seven of the eight herbs (*Fructus Citri Sarcodactylis* being the exception) had demonstrated strong anti-inflammatory, antioxidant and anti-cancer activities [15–17]. Similar pharmacological properties of ursolic acid, quercetin, epicatechin and lauric acid that were present in *Radix Asparagi*, *Radix Actinidia chinensis Planch, Fructus Hippophae* and *Fructus Citri Sarcodactylis*, respectively, had also been demonstrated [18–21].

### Generating compound-putative target network for TFS

Generally, the effectiveness of the TCM formulas to prevent and control complex diseases depends on the synergistic effects between multiple compounds and their targets [22]. Therefore, we explored the therapeutic targets of the predicted active compounds of TFS. Towards this, we integrated the available chemical, genomic and pharmacological information to predict putative targets of the candidate compounds [10, 64]. We obtained 468 putative targets for 113 of the 136 candidate compounds whereas the 23 others did not have any corresponding targets (Supplementary Table 2). The numbers of putative targets in RPQ, VB, RA, BII, RAO, RACP, FH and FCS, were 150, 75, 198, 86, 213, 220, 404 and 69, respectively (Supplementary Table 3). Although the numbers of targets in each herb were different, they overlapped dramatically in the 8 herbs (Supplementary Table 4). In other words, different ingredients in TFS shared common or similar targets with synergistic effects . For instance, both *Radix Panacis Quinquefolii* and *Fructus Citri Sarcodactylis* were able to inhibit tumorigenesis by reducing inflammation and proliferation related genes and proteins that acted synergistically, including iNOS, COX-2, Bcl-2, cyclin D1 [23, 24].

To gain insights into the role of the putative targets involved in various biological processes and molecular functions, we performed preliminary GO (Gene Ontology) analysis with Omicsbean, a commercial database based on DAVID (the Database for Annotation, Visualization and Integrated Discovery) [25] and found that the putative targets were enriched in cell cycle, apoptosis, inflammation, angiogenesis and cellular metabolism, all of which are the hallmarks of cancer (Supplementary Figure 2).

**Figure 2:**
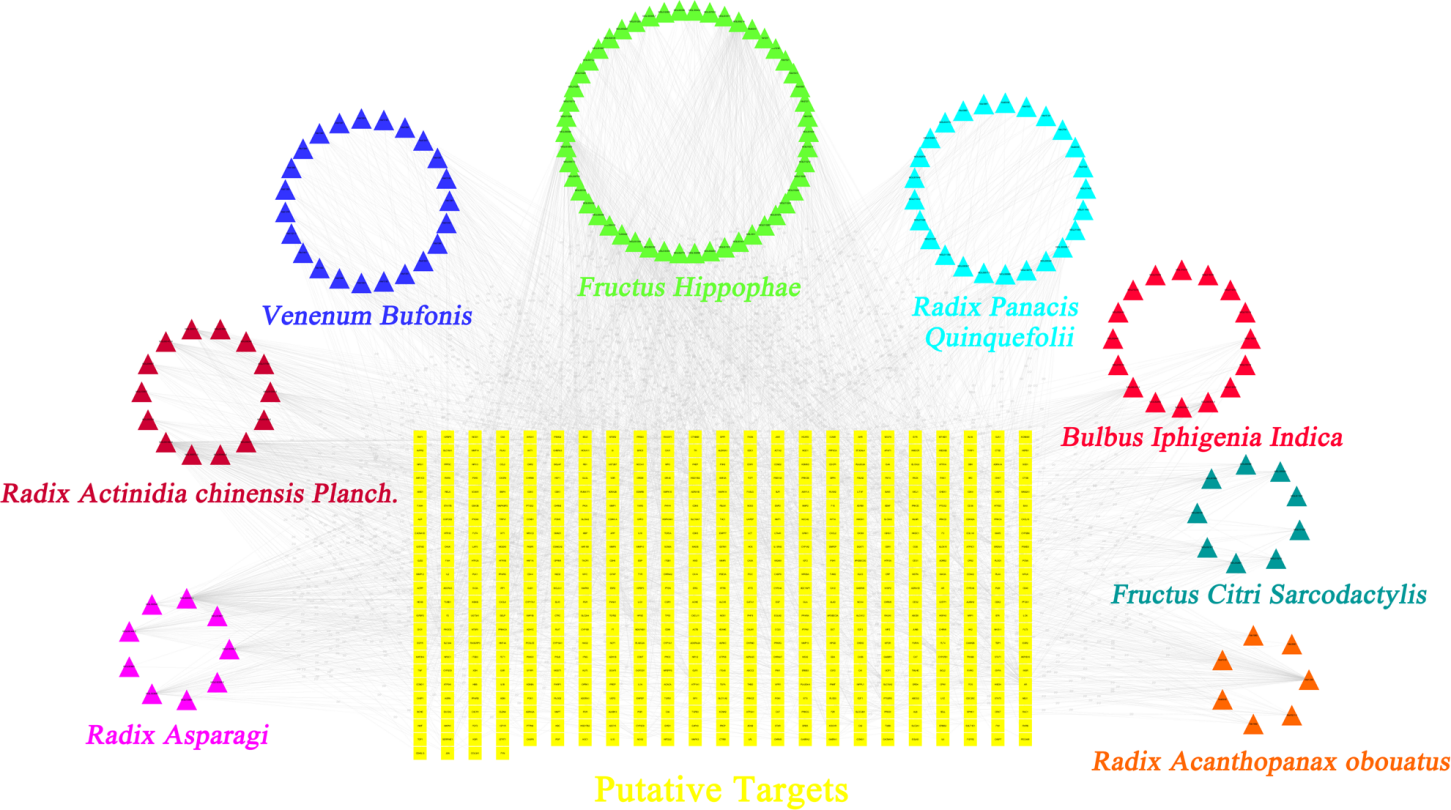
Construction of the TFS compound-putative target network The compound-putative target network was constructed by linking the candidate compounds and their putative targets of the 8 herbs (*Radix Panacis Quinquefolii*, *Venenum Bufonis*, *Radix Asparagi*, *Bulbus* *Iphigenia Indica*, *Radix Acanthopanax obouatus*, *Radix Actinidia chinensis Planch*., *Fructus Hippophae* and *Fructus Citri Sarcodactylis*), which are constituents of TFS. The nodes representing candidate compounds are shown as polychrome triangles and the targets are indicated by yellow squares.

Further, to understand the complex interactions between the compounds and their corresponding targets at a system level, we constructed a network based on the candidate compounds of TFS and their potential targets (Figure 2 and Supplementary Table 5). The network contained 631 nodes and 2985 compound-target interactions of which sixty-five candidate compounds had a median of eleven degrees (number of related targets) suggesting that most compounds influenced multiple targets. Specifically, we found that quercetin, kaempferol and progesterone acted on 162, 71 and 112 targets, respectively, and therefore could be the crucial pleiotropically active compounds for TFS owing to their crucial positioning in the network. Previously, several studies demonstrated that quercetin inhibited inflammation, enhanced immune function and promoted apoptosis by modulating key pathways regulating cancer, such as, prostaglandin G/H synthase 2, interleukin (IL)-2, peroxisome proliferator activated receptor gamma and caspase-9 [26, 27].

### Identification of candidate targets for TFS against CRC

**Figure 3:**
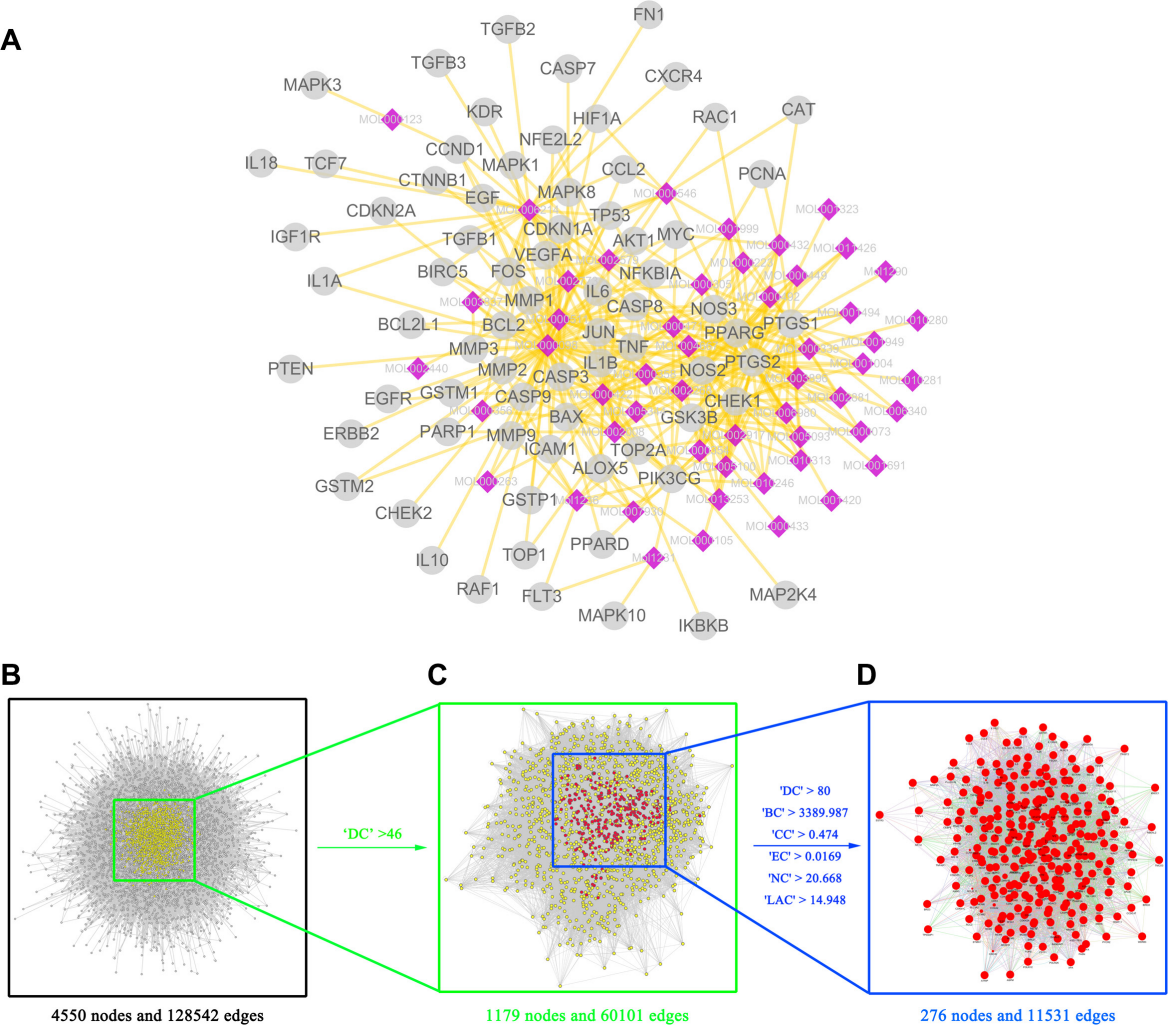
Identification of candidate targets for TFS against CRC (**A**) TFS shared 78 putative targets with known anti-CRC drugs. The compound-putative target network was constructed by linking the overlapped targets (between TFS putative and known CRC-related) and the homologous candidate compounds of TFS. The nodes representing candidate compounds are shown as polychrome rhombus and the targets are presented as grey circles. (**B**) The interactive PPI network of TFS putative targets and known CRC-related targets made of 4550 nodes and 128542 edges is shown. (**C**) PPI network of significant proteins extracted from (B). This network is made of 1179 nodes and 60101 edges. (**D**) PPI network of candidate TFS targets for CRC treatment extracted from (C). This network is made of 276 nodes and 11531 edges.

Since the use of a drug is based on its targets, we collected 446 CRC-related targets from five existing resources, namely, the DrugBank database, Online Mendelian Inheritance in Man (OMIM) database, the Genetic Association Database (GAD), the Kyoto Encyclopedia of Genes and Genomes (KEGG) Pathway Database and the Therapeutic Target Database (TTD) (Supplementary Table 6). Our analysis showed that the 8 herbs of TFS shared 78 putative targets with those of known anti-CRC drugs showing the possible therapeutic targets in this formula (Figure 3A and Supplementary Table 7).

System biology studies have shown that cancer genes and proteins are interconnected and the protein-protein interaction (PPI) networks are relevant to understand the role of various proteins in complex diseases like cancer [52, 53]. Therefore, we constructed a putative target network (8631 nodes and 191590 edges) and a known CRC-related target network (7339 nodes and 142492 edges) using the PPI data. Further, to unravel the pharmacological mechanisms of TFS against CRC, we intersected the two networks consisting of 4550 nodes and 128542 edges (Figure 3B). Based on a previous study of Li and others [52], we identified nodes with degrees that were more than twice the median degree (23) of all nodes as significant targets. Thus, we constructed a network of significant targets for TFS against CRC that had 1179 nodes and 60101 edges (Figure 3C). We further selected seven topological features to identify candidate targets, namely, ‘degree centrality (DC)’, ‘betweenness centrality (BC)’, ‘closeness centrality (CC)’, ‘eigenvector centrality (EC)’, ‘network centrality (NC)’ and ‘local average connectivity (LAC)’, that were based on a plugin named CytoNCA [54]. Based on the median values for ‘DC’, ‘BC’, ‘CC’, ‘EC’, ‘NC’ and ‘LAC’ that were 80, 3389.987, 0.4740185, 0.016892, 20.66752 and 14.94827, respectively, we identified 276 candidate targets with ‘DC’ > 80, ‘BC’ > 3389.987, ‘CC’ > 0.474, ‘EC’ > 0.0169, ‘NC’ > 20.668 and ‘LAC’ > 14.948 (Figure 3D). Detailed topological features and the PPI network are shown in Supplementary Table 8 and Figure 3B–3D, respectively.

### Enrichment analysis of candidate targets for TFS against CRC

**Figure 4:**
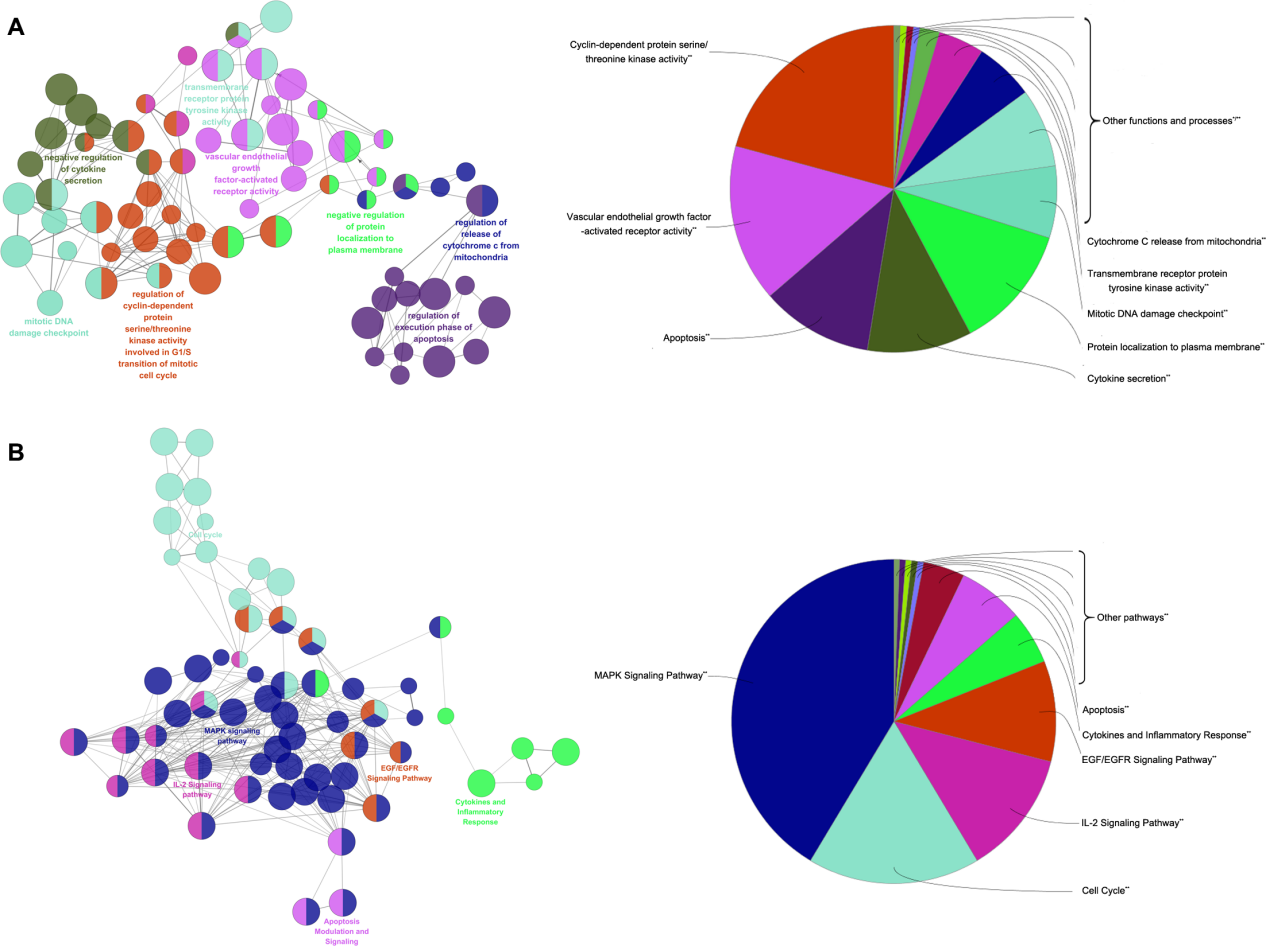
Enrichment analysis of candidate targets for TFS against CRC The enrichment analysis is represented by the pie charts (right) as generated by ClueGo and the most vital term in the group is labeled (left). Functionally related groups partially overlap. Representative enriched pathway (*P* < 0.05) interactions among main TFS targets. (**A**) Candidate TFS targets enriched in the representative molecular function/biological process are shown. (**B**) Candidate TFS targets enriched in the representative signalling pathway are shown.

To further clarify the possible roles of the 276 candidate targets and investigate the relationship between the functional groups and their underlying scientific annotations in the biological networks, we used a Cytoscape plugin, ClueGO [55]. The results were classified into two categories, namely, molecular functions/biological processes and the signaling pathway (Figure 4 and Supplementary Tables 11, 12). Specifically, we obtained molecular functions/biological processes related with activation of CDKs, transmembrane receptor protein tyrosine kinase and VEGFR, apoptosis, cytokine secretion, DNA damage checkpoint and cytochrome C release (Figure 4A). The signaling pathways were MAPK, EGF/EGFR and cell cycle, apoptosis and inflammation-related (Figure 4B). Based on these data, we postulated that TFS inhibited CRC by regulating the major signaling pathways involved in CRC-related pathology, namely, cell proliferation, apoptosis, chronic inflammation and angiogenesis. Therefore, to validate the effects of TFS, we performed molecular biological assays *in vitro* and *in vivo*.

### Experimental validation

### TFS inhibited CRC growth partially by arresting the cell cycle

**Figure 5:**
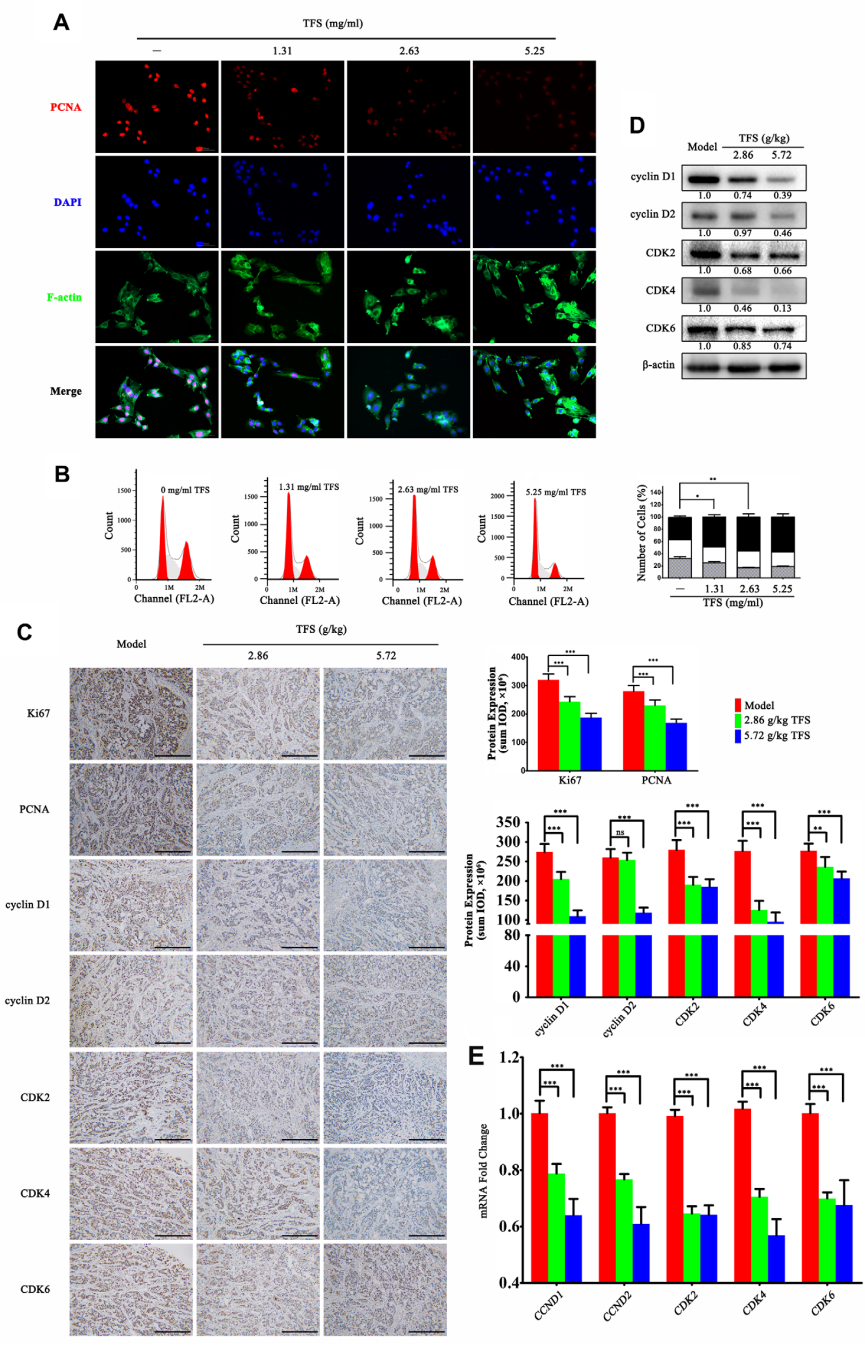
TFS induces cell cycle arrest of CRC cell lines (**A**) HT-29 cells were treated with or without TFS (1.31, 2.63 and 5.25 mg/ml) for 24 h. The immunofluorescence staining of PCNA was performed as described in Materials and Methods (200×, blue, DAPI; red, PCNA; green, F-actin). (**B**) HT-29 cells were exposed to 1.31, 2.63 and 5.25 mg/ml TFS or culture medium only for 24 h and then stained with Propidium iodide for detecting the cell cycle by FACS (left). The quantitative data of the cell cycle analysis (G0/G1, S, and G2/M phases) based on an average of triplicate experiments is shown as mean ± SD (right). *P < 0.05, ^*^P < 0.01 (versus negative control). (**C**) IHC analysis of Ki67, PCNA, cyclin D1, cyclin D2, CDK2, CDK4 and CDK6 expression in HT-29 transplanted tumor tissue in different treatment groups is shown (200×, scale bar represents 500 μm; left). The quantitative analysis of IHC data is shown as mean ± SD (right). ^*^P < 0.01, ^**^*P < 0.001 (versus model) (**D**) The protein expression of Ki67, PCNA, cyclin D1, cyclin D2, CDK2, CDK4 and CDK6 as determined by western blotting is shown. Quantifcation of protein level was normalized to β-actin using densitometry. (**E**)The mRNA levels of CCND1, CCND2, CDK2, CDK4 and CDK6 in HT-29 transplanted tumor tissue in different treatment groups as determined by real-time qPCR is shown. GAPDH was used as the loading control. The data are presented as mean ± SD. ^**^*P < 0.001 (versus model).

Deregulated and excessive proliferation is one of the hallmarks of all cancers including CRC [33, 34]. Therefore, we investigated the effects of TFS on the proliferation of HT-29 cells *in vitro*. As shown in Figure 5A, we observed decreased expression of PCNA (proliferating cell nuclear antigen) in a dose-dependent manner in the TFS-treated group. Further, we evaluated the effects of TFS on the cell cycle by staining the cells with propidium iodide (PI) after treatment with different doses of TFS (1.31, 2.63 and 5.25 mg/ml) for 24 h along with appropriate controls and performed FACS analysis. As shown in Figure 5B, treatment with increasing doses of TFS increased the average percentages of HT-29 cells in the G0/G1-phase from 37.54% to 58.61%, suggesting that TFS arrested cell cycle. To ascertain the cell proliferation impact of TFS *in vivo*, we performed immunohistochemical assays for cell cycle markers, PCNA and Ki67 in the transplanted HT-29 tumors and found similar results (Figure 5C). Since TFS induced cell-cycle arrest in the G0/G1 phase, we analyzed the expressions of cell cycle regulatory proteins associated with G1/S phase transition (cyclin D1, cyclin D2, CDK2, CDK4 and CDK6). Based on western blot (Figure 5D), IHC assay (Figure 5C) and quantitative PCR (Figure 5E), we found that their expression decreased in a concentration-dependent manner in the TFS-treated groups compared with the model group. Hence, we concluded that TFS inhibited CRC by inducing cell-cycle arrest through its candidate targets including CDKs and cyclin Ds.

### TFS induced apoptosis in CRC cells

**Figure 6:**
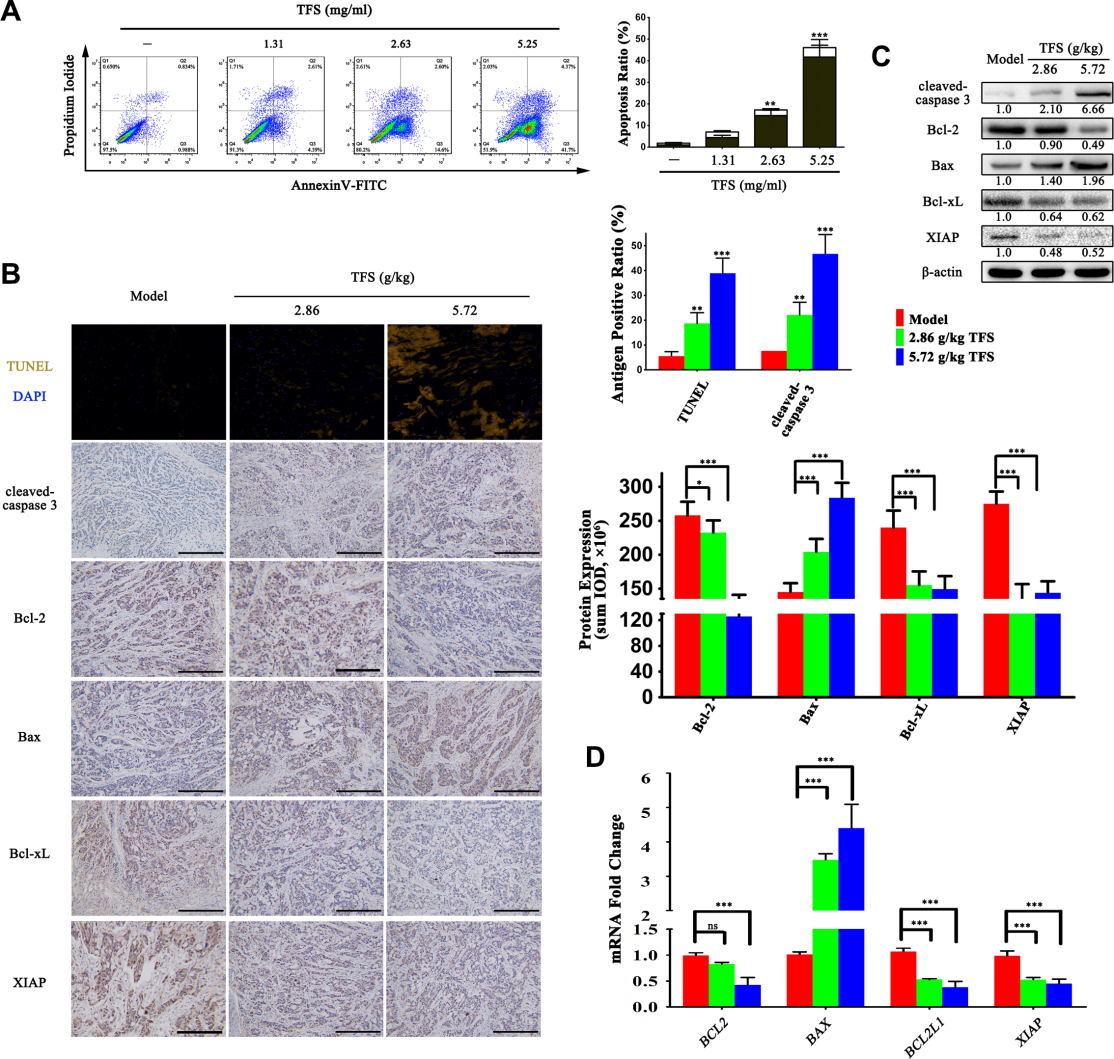
TFS induces apoptosis in CRC cells (**A**) The HT-29 cells were exposed to 1.31, 2.63 and 5.25 mg/ml TFS or culture medium for 48 h and stained with propidium iodide and AnnexinV-FITC for detecting the apoptosis by flow cytometry (left). The percentages of apoptotic cells (the lower-right quadrant of the fluorescence-activated cell sorting histograms (percentage of early apoptotic cells) and the upper-right quadrant (percentage of late apoptotic cells)) are shown (right). The data represent mean ± SD. ^*^P < 0.01, ^**^*P < 0.001 (versus negative control). (**B**) The TUNEL and IHC analysis of cleaved-caspase 3 expression that denotes the level of apoptosis in HT-29 transplanted tumor tissue in different treatment groups (left). The protein expression level of apoptosis-related protein (Bcl-2, Bax, Bcl-xL and XIAP) that were detected by IHC assay (200×, scale bar represents 500 μm) are shown (left). The quantitative analysis of TUNEL and IHC data is shown as mean ± SD (right). *P < 0.05, ^*^P < 0.01, ^**^*P < 0.001 (versus model). (**C**) The protein expression of cleaved-caspase 3, Bcl-2, Bax, Bcl-xL and XIAP were measured by western blotting. Quantifcation of protein level was normalized to β-actin using densitometry. (**D**) The mRNA levels of these aforesaid genes in HT-29 transplanted tumor tissue in the different treatment groups were determined by real-time qPCR. GAPDH was used as the loading control. The data are presented as mean ± SD. ^**^*P < 0.001 (versus model).

In tandem with enhanced over-proliferation, dysregulation of apoptosis is a key feature of CRC due to aberrant activities and expressions of apoptosis-related molecules [38, 39]. Many of the candidate targets that we identified in our analysis were also associated with regulation of cell apoptosis. Therefore, to determine if TFS promoted CRC apoptosis, we double-stained HT-29 cells with AnnexinV/PI and performed FACS analysis. We observed that higher doses of TFS induced greater apoptotic rates than the control group (*p* < 0.05; Figure 6A). To confirm the effects of TFS on apoptosis *in vivo*, tumors from CRC xenograft mice were examined by using cleaved caspase-3 IHC staining and TUNEL (terminal deoxynucleotidyl transferase-mediated deoxyUTP-fluorescein nick end labeling) assay. We found that TFS significantly increased the number of cells with cleaved caspase-3 and positive TUNEL staining suggesting enhanced apoptosis (Figure 6B). To further explain the mechanism by which TFS facilitated apoptosis, we performed RT-PCR, Western blot and IHC assay to examine the mRNA and protein expressions of apoptosis-related proteins, namely, Bcl-2, Bax, Bcl-xL and XIAP, in transplanted CRC tumors with or without TFS treatment. We found that TFS treatment significantly reduced the mRNA expression of anti-apoptotic proteins, Bcl-2, Bcl-xL and XIAP and elevated pro-apoptotic Bax levels (Figure 6D). The IHC staining and western blot analysis further confirmed enhanced expression of the pro-apoptotic protein Bax and reduced expression of the anti-apoptotic proteins, Bcl-2, Bcl-xL and XIAP (Figure 6B, 6C). In conclusion, TFS promoted apoptosis in CRC cells by upregulating pro-apoptotic proteins and downregulating the anti-apoptotic proteins like Bcl-2 and Bax.

### TFS inhibits aberrant angiogenesis upregulated during CRC progression

**Figure 7:**
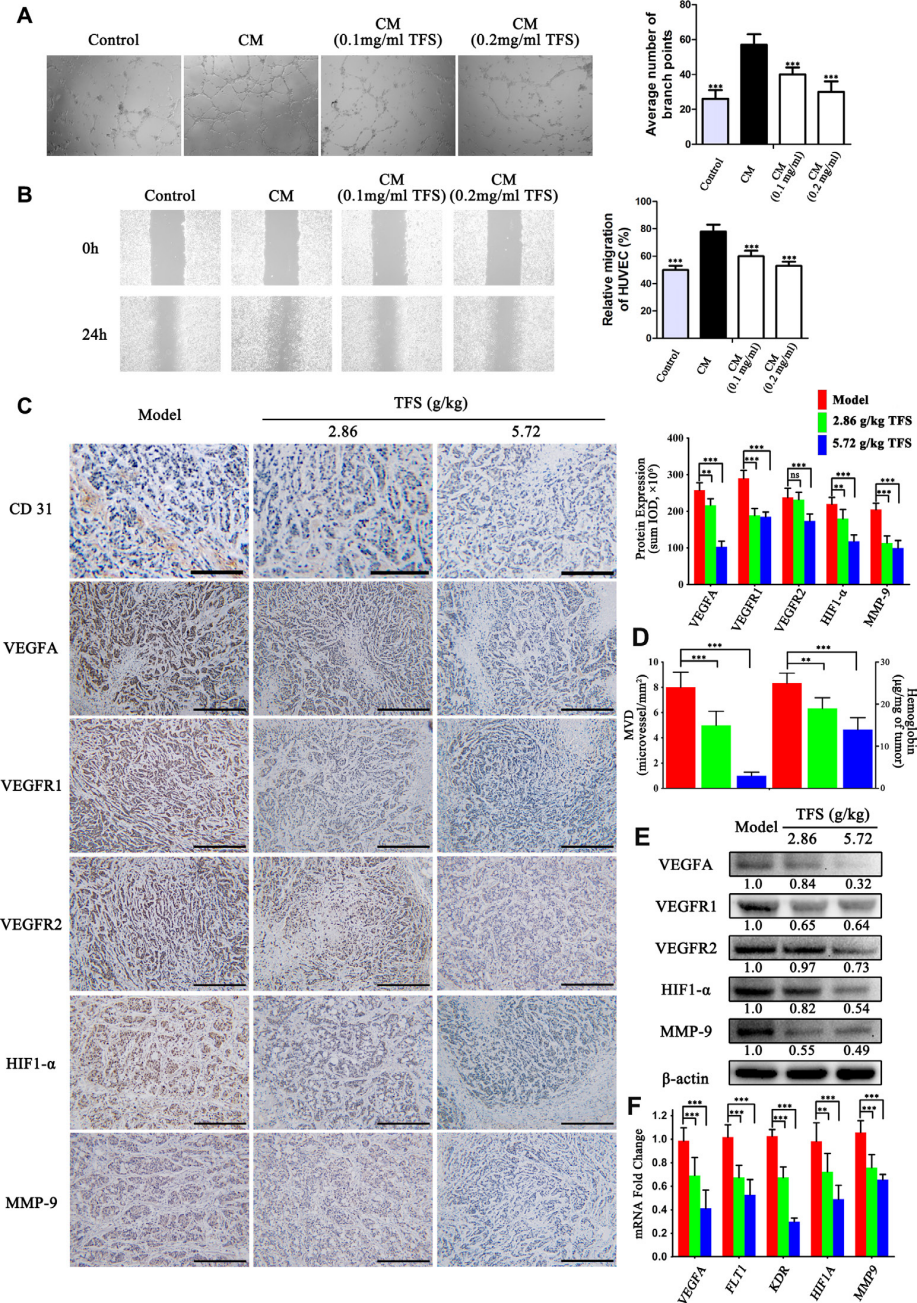
TFS normalizes aberrant angiogenesis during CRC progression (**A**) HUVEC cells were incubated with CM (condition medium) from HT-29 for 24 h in the presence or absence of TFS. Then, the capillary-like tubeformation and tubular structures of HUVEC were determined and photographed (40×). The vascular branch of each groups was quantified. The data are presented as mean ± SD. ^**^*P < 0.001 (versus CM). (**B**) Representative images (40×) of wound healing experiments with HUVEC monolayers in were exposed to CM from HT-29 (treated with or without TFS) for 24 h. The data are presented as mean ± SD. ^**^*P < 0.001 (versus CM). (**C**) IHC analysis of CD31, VEGFA, VEGFR1, VEGFR2, HIF1-α and MMP-9 expression in HT-29 transplanted tumor tissue in different treatment groups (200×, scale bar represents 500 μm) is shown (left). The quantitative analysis of IHC data are shown as mean ± SD (right). ^*^P < 0.01, ^**^*P < 0.001 (versus model). (**D**) The level of intratumoral microvessel density (represented by CD31 immuno-reactivity) and hemoglobin concentration in HT-29 transplanted tumor tissue. The data are presented as mean ± SD. ^*^P < 0.01, ^**^*P < 0.001 (versus model). (**E**) The protein expression of VEGFA, VEGFR1, VEGFR2, HIF1-α and MMP-9 were determined by western blotting. Quantifcation of protein level was normalized to β-actin using densitometry. (**F**) The mRNA levels of these above genes in HT-29 transplanted tumor tissue were determined by real-time qPCR. GAPDH was used as the loading control. The data are presented as mean ± SD. ^*^P < 0.01, ^**^*P < 0.001 (versus model).

A critical process during CRC progression is angiogenesis and our previous data showed a large number of candidates that have inhibitory effect on angiogenesis [43, 44]. To investigate the effects of TFS on the angiogenesis pathway, we collected serum-starved conditioned media from HT-29 cells treated with or without TFS and stimulated endothelial cell tube formation and migration using HUVECs. We observed that media from TFS treated cancer cells significantly reduced CRC cell-induced endothelial branching and migration (Figure 7A and 7B). In addition, we assessed the the microvessel density in transplanted CRC tumors by CD31 immuno-reactivity and hemoglobin concentration and found that the TFS treatment group decreased intra-tumoral microvessel density and hemoglobin concentration in comparison to the PBS control group (*p* < 0.05) suggesting the anti-angiogeneic effect of TFS (Figure 7D). Furthermore, we examined proteins involved in angiogenesis like VEGF, and their receptors. Based on the IHC staining assay, the levels of VEGF-A in TFS treated tumor tissues was diminished compared to those in the model group (Figure 7C). Similarly, mRNA and protein levels of VEGFR1 and VEGFR2 were lower in the TFS treated tumors in comparison to the control as analyzed by RT-PCR, western blot and IHC assays (Figure 7C, 7E, and 7F). We also observed downregulation of both HIF1-α and MMP-9 mRNA and proteins in TFS treated tumors (Figure 7C, 7E, and 7F). Overall, these data indicated that TFS treatment effectively inhibited the angiogenic response in CRC and confirmed the outcomes of the functional enrichment analysis.

### TFS suppressed inflammation in CRC neoplastic tissue

**Figure 8:**
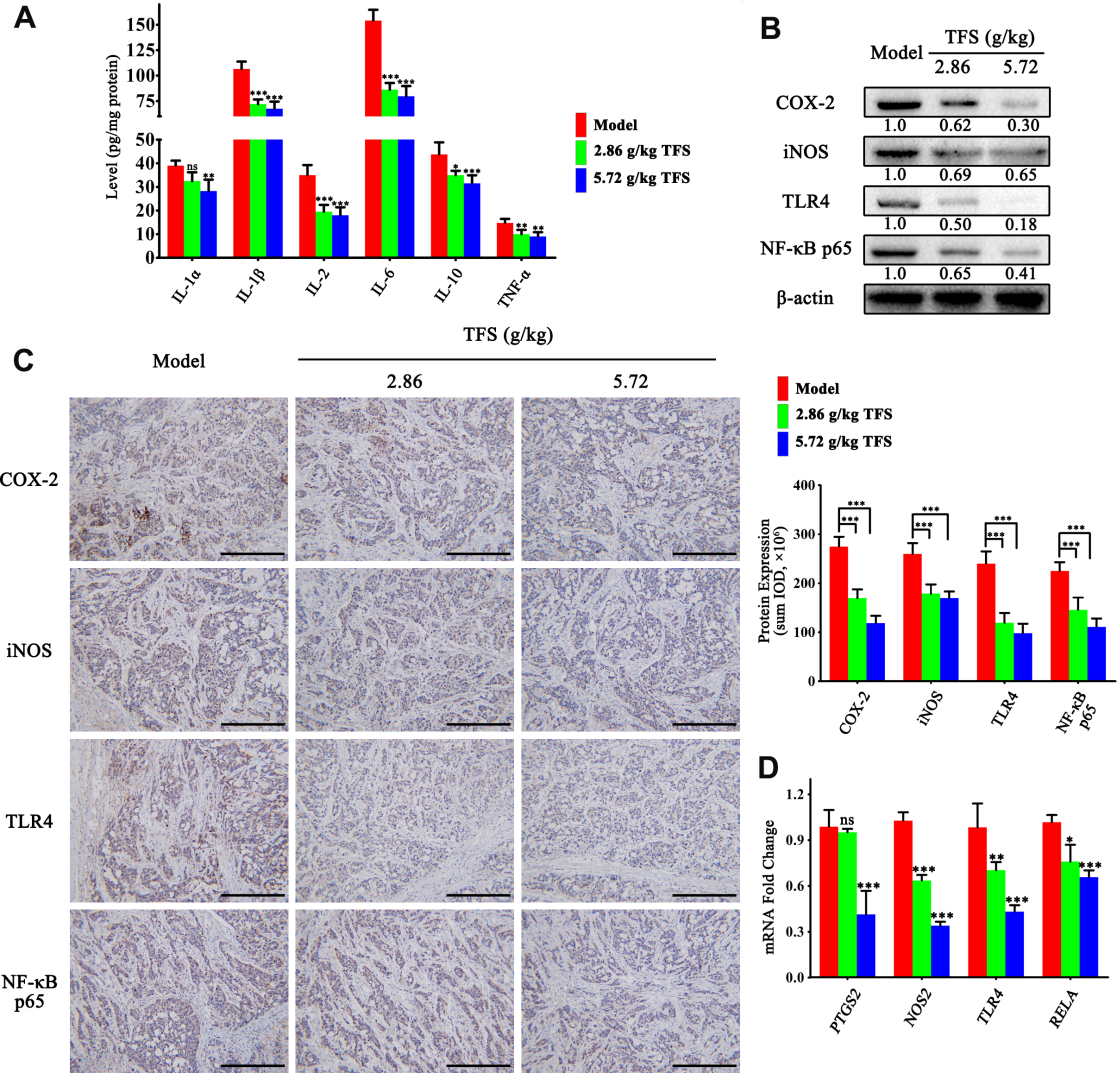
TFS inhibits chronic inflammation in CRC neoplastic tissue (**A**) ELISA analysis of IL-1α, IL-1β, IL-2, IL-6, IL-10 and TNF-α expression in HT-29 transplanted tumor tissue in different treatment groups is shown. The data are presented as mean ± SD. *P < 0.05, ^*^P < 0.01, ^**^*P < 0.001 (versus model). (**B**) The expression of pro-inflammatory mediators (COX-2 and iNOS) and vital signalling proteins (TLR4 and p65) were measured by western blotting. Quantifcation of protein level was normalized to β-actin using densitometry. (**C**) The protein level of the above proteins as detected by IHC assay (200×, scale bar represents 500 μm; Left). The quantitative analysis of the IHC data are shown as mean ± SD (right). ^**^*P < 0.001 (versus model). (**D**) The mRNA levels of these aforementioned genes in HT-29 transplanted tumor tissue were analyzed by real-time qPCR. GAPDH was used as the loading control. The data are presented as mean ± SD. *P < 0.05, ^*^P < 0.01, ^**^*P < 0.001 (versus model).

The risk of CRC can be reduced by using anti-inflammatory non-steroidals and cyclooxygenase-2 selective inhibitor celecoxib that demonstrate the central role of chronic inflammation in the cancer [49, 50]. As shown in Figure 8A, the TFS treated xenograft tumors showed lower expression of IL-1α, IL-1β, IL-2, IL-6, IL-10 and TNF-α in comparison to the controls (Figure 8A). In addition, we observed lower protein and mRNA levels of pro-inflammatory mediators, COX-2 and iNOS (Figure 8B–8D). We further analyzed the TLR4/NF-κB signaling pathway and found that TFS treatment inhibited mRNA and protein expression of TLR4 and p65 in comparison to the controls in a dose-dependent manner (Figure 8B–8D). These data further corroborated our previous findings that TFS treatment significantly reduces chronic inflammation associated with CRC progression.

**Figure 9:**
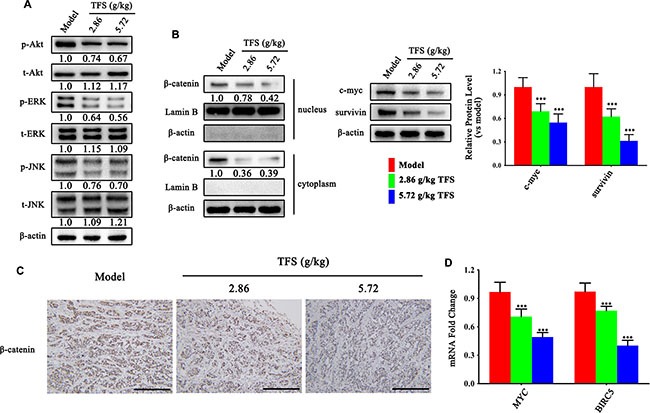
TFS restrains hyperactive Wnt/β-catenin and MAPK signaling pathways in CRC (**A**) The activity of MAPK (ERK and JNK) signalling pathway was measured by western blotting. Quantifcation of protein level was normalized to β-actin using densitometry. (**B**) The nuclear levels of β-catenin and the protein levels of its downstream targets (c-myc and survivin) as detected by western blotting are shown (left). The quantitative analysis of WB data is shown as mean ± SD (right). ^**^*P < 0.001 (versus model). Quantifcation of protein level was normalized to β-actin or Lamin B using densitometry. (**C**) The IHC analysis showing expression of β-catenin is shown (200×, scale bar represents 500 μm). (**D**) The mRNA levels of MYC and BIRC5 in HT-29 transplanted tumor tissue were analyzed by real-time qPCR. GAPDH was used as the loading control. The data are presented as mean ± SD. ^**^*P < 0.001 (versus model).

We also investigated hyperactive Wnt/β-catenin and MAPK signaling pathways that are upregulated in CRC. As shown in Figure 9A, we observed a dose dependent suppression of ERK and JNK activities by TFS. Moreover, the protein expression and nuclear location of β-catenin (Figure 9B, 9C), as well as the mRNA levels of its downstream targets, c-myc and survivin (Figure 9B, 9D), decreased in the TFS-treated groups compared to the control group.

**Figure 10:**
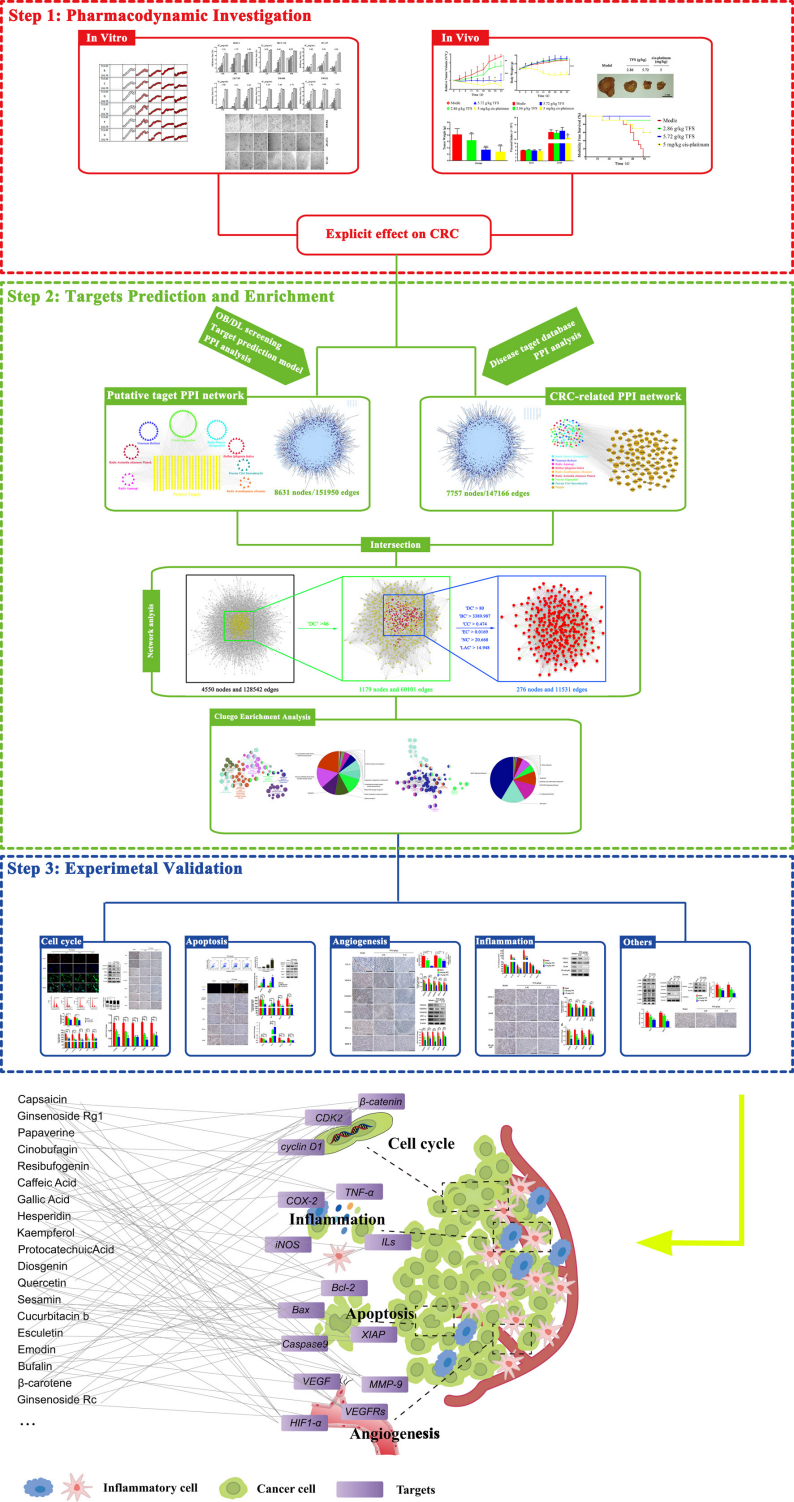
The schematic diagram of the research methodology and the proposed model of Tianfoshen oral liquid acting on colorectal cancer are shown

Collectively, TFS had significant therapeutic effect on multiple critical pathological pathways of CRC like cell proliferation, apoptosis resistance, angiogenesis and chronic inflammation by regulating key targets involved in carcinogenesis (Figure 10) and thereby showcased its efficacy in treating CRC.

## DISCUSSION

Colorectal cancer (CRC) is a disease that originates from the epithelial cells lining the rectum or the colon and is frequently associated with genetic alterations in the K-ras and β-catenin oncogenes as well as APC and Bax tumor suppressors [28–32]. These mutated genes have a number of downstream target genes that are involved in crucial cellular processes like cell cycle, proliferation, metabolism, inflammation. Eventually, this results in establishment of CRC and its progression. Given the involvement of multiple pathological processes in cancer, drugs that interfere with tumorigenesis are prospective treatments for CRC and the traditional Chinese medicines (TCM) are one of them.

In this study, we first confirmed that Tianfoshen oral liquid (TFS), a clinical TCM formula that is used for NSCLC treatment was effective in inhibiting CRC, both *in vitro* and *in vivo*. However, given the complexity of active components in TFS and the diversity of potentialmodulated targets in human body, it was difficult to characterize the scientific basis and underlying pharmacological mechanisms of TFS for CRC by conventional methods. Therefore, we utilized a newly developed pharmacological approach to analyze the active compounds and therapeutic targets of TFS. This involved construction and analysis of a target network based on the prediction of candidate compounds and speculation of multiple drug targets. Having identified candidate targets based on the network, we further annotated by functionally analysis using ClueGO. This analysis highlighted that TFS inhibited CRC by regulating aberrant cell proliferation, apoptosis resistance, induced angiogenesis-related signaling and chronic inflammation that were induced by CRC-related oncogenes and dysregulated suppressor genes.

Aberrant cancer cell proliferation is associated with mutations in oncogenes and tumor suppressor genes like β-catenin and APC and their downstream signaling cascades including the MAPK pathway [33, 34]. Ursolic acid, an active compound produced by *Radix Actinidia chinensis Planch* and *Fructus Hippophae*, has been shown to suppress the proliferation of CRC cells by facilitating N-terminal phosphorylation and subsequent proteasomal degradation of β-catenin [35]. Moreover, the extracts of *Radix Panacis Quinquefolii*, *Venenum Bufonis*, *Radix* *Actinidia chinensis Planch*, *Fructus Hippophae* and *Fructus Citri Sarcodactylis* induce cell-cycle arrest and inhibit cell proliferation [36, 37]. As shown in Supplementary Table 8, TFS targets included cell cycle-related proteins (CDK, c-Myc), oncogene (β-catenin) and their downstream signaling pathway proteins (ERK, JNK, MEK) which indicated that the anti-proliferative activity of TFS was in good agreement with previous literature investigations [35–37].

Altered apoptosis is central to CRC progression. Previously, abnormal expression and activity of anti-apoptosis-related proteins and genes including the inhibitor of apoptosis protein family, the Bax family and the BH3-only protein family has been associated with poor prognosis and drug resistance in CRC [38, 39]. Composite compounds and extractive fractions of *Radix Panacis Quinquefolii* and *Venenum Bufonis* can induce the apoptosis of CRC cells *in vitro* and *in vivo* [40, 41]. Additionally, quercetin, as an active compound shared by *Radix Asparagi*, *Radix Actinidia chinensis Planch*. and *Fructus Hippophae*, can significantly suppress the activity of viable CRC cells and then induce programmed deaths [42]. Consistently, apoptosis-related proteins (Bax, Bcl-2, Bcl-x, the caspase family, PPAR), related oncogene (β-catenin) and their downstream signaling pathway (PI3K/Akt), as the candidate targets of 8 herbs in TFS (Supplementary Table 8), may represent the apoptosis-promoting effect of the formula and also be eligible therapeutic targets for treating CRC.

Another crucial factor in CRC progression is angiogenesis. Angiogenesis-related proteins and factors are over-expressed and activated in CRC and are potential therapeutic targets for CRC [43, 44]. Previously, the active compounds of *Radix Panacis Quinquefolii*, *Bulbus Iphigenia Indica* and *Radix Actinidia chinensis Planch*. (example., ginsenoside Re, tubeimoside and quercetin) have demonstrated anti-angiogenic effects [45–48]. Our study documented and verified the inhibitory effect of TFS on pro-angiogenic proteins (e.g. VEGFs, VEGFRs and MMPs) involved in CRC, both *in vivo* and *in vitro*.

Further, the chronic inflammatory bowel disease involves interactions between chemokines, cytokines, immune cells, inflammatory cells and pro-inflammatory mediators that promote the proliferation, growth and invasion of tumor cells [49]. Thus, targeting chronic inflammation is important in cancer therapy [50]. Many candidate targets of TFS shown in Supplementary Table 8 like cytokines (IL-1, IL-6, IL-10 and TNF-α), chemokines (CCL3, CCL5), chemokine receptor (CXCR4), pro-inflammatory mediators (COX-2, LOX-5 and iNOS) and related signaling pathways (NF-κB, Nrf2) participated in CRC progression. Especially, *Radix Panacis Quinquefolii*, the predominant component of this formula, can inhibit AOM/DSS-induced CRC by regulating the expressions and activations of pro-inflammatory cytokines [51].

On this basis, we conduct a wide range of experiment to further elucidated the mechanisms of by which TFS exerted therapeutic effects on CRC. Combining literature investigation, enrichment analysis and experimental validation, we postulated that TFS may treat CRC through activating/inhibiting the levels of its targets which play pivotal roles in cancer proliferation, apoptosis, angiogenesis and inflammation in a multi-component, multi-target and multi-link manner.

Despite these important discoveries, this study has limitations. Firstly, although TFS inhibited colorectal cancer in a preclinical study with no side effects with similar efficacy as the first-line drug of colorectal carcinoma chemotherapy, oxaliplatin, the effects of TFS were unclear and needed further extensive analysis in clinical trials. Secondly, the effects of some composite compounds of the herbs used in TFS may have been neglected due to incomplete information. Thirdly, it is difficult to directly teasing out specific targets and key active components from a chemically complex and cognitively scarce TCM formula. In the current experimentation, we mainly focused on the pathological processes mitigated by TFS and the related key targets for CRC. Therefore, future studies are necessary to clarify the role of vital individual components of TFS on the pathological process and the specific targets. In future, there is a need to develop collaborative therapeutic regimens for the clinic that targets the several synergistic pathways that maintain the malignant state of CRC and formula like TFS may be beneficial.

## MATERIALS AND METHODS

### Chemicals

Dulbecco's modified Eagle's medium, RPMI 1640 medium, Leibovitz's L-15 medium, Fetal bovine serum (FBS), trypsin-EDTA and penicillin/streptomycin were purchased from Gibco (Thermo Fisher Scientific, USA). Antibodies against PCNA, CDK2, CDK4, CDK6, cyclin D1, cyclin D2, cyclin D3, Ki67, CD31, VEGFR1, VEGFR2, EGFR, MMP3, MMP9, HIF-1-alpha, COX2, LOX, iNOS, JNK, ERK1/2, JNK, phospho-ERK (T202/T204), phospho-JNK (T183/Y185), beta-catenin, Bcl-2, Bax, Bad, Bcl-xL and XIAP were provided by CST (Cell Signaling Technology). Unless otherwise noted, all other materials were obtained from Sigma-Aldrich.

### Chemical composition of Tianfoshen oral liquid

The chemical composition of all the eight herbs that constitute Tianfoshen oral liquid (TFS) was obtained from the Chinese Academy of Sciences Chemistry Database (http://www.organchem.csdb.cn/, last updated: November 18, 2015) and the Traditional Chinese Medicine System Pharmacology Database (http://tcmspnw.com/, version: 2.0) [14]. The information regarding the 542 chemicals is reported in Supplementary Table 9 and it includes 153 from *Radix Panacis Quinquefolii*, 24 from *Venenum Bufonis*, 32 from *Radix Asparagi* , 43 from *Bulbus Iphigenia Indica*, 7 from *Radix Acanthopanax obouatus*, 26 from *Radix Actinidia chinensis Planch*., 226 from *Fructus Hippophae* and 81 from *Fructus Citri Sarcodactylis* , respectively.

### Candidate compounds screening parameters

We estimated OB prescreening that indicates the degree of an oral dose of drug that distributes to the bloodstream [56] and drug-likeness that evaluates the structural similarity between compounds and the clinical drugs in the Drugbank database [57] according to calculations set-up by Wang and others [58]. We defined OB of 30% and drug-likeness index of 0.18 to select candidate compounds [10]. Some chemical compounds like bufalin, cinobufotalin and resibufogenin did not obtain an accurate DL index and the set minimum OB as 35%. Others such as ginsenoside Rb2, gamabufogenin, 19-oxobufalin, gracillin, cucurbitacin b and sesamin, were initially omitted based on the screening rules [59–63]. However, we manually retrieved these candidate compounds for further analysis as their anti-cancer properties have been previously reported.

### Identifying CRC related targets in TFS

For the current study, we chose the systematic drug targeting approach developed by Wang and others to identify potential targets for medicinal composition of TFS [64]. Known CRC-related targets were obtained from five existing resources: (1) The DrugBank database (http://www.drugbank.ca/, version: 4.3): We used interactions between FDA-approved drugs for CRC treatment and human genes/proteins-derived targets and obtained 17 known CRC-related targets [65]; (2) The OMIM database (Online Mendelian Inheritance in Man; http://www.omim.org/): We screened with a keyword ‘‘colorectal cancer’’ and found 16 known CRC-related targets [66]; (3) Genetic Association Database (GAD; http://geneticassociationdb.nih.gov/): We searched with the keyword ‘‘colorectal cancer’’ and obtained 370 known targets that change during CRC [67]; (4) The Kyoto Encyclopedia of Genes and Genomes (KEGG) Pathway Database (http://www.genome.jp/kegg/): We obtained 59 known CRC-related targets in the CRC pathway (KEGG ID: map 05210) [68]; and (5) The TTD database (http://database.idrb.cqu.edu.cn/TTD/): We searched with the keyword “colorectal cancer” and obtained 38 known targets related with CRC [69]. Detailed information of these known therapeutic targets is described in Supplementary Table 6. After redundancy was deleted, 446 known CRC-related targets were finally collected. Further, PPI data was obtained using the Cytoscape plugin Bisogenet and analyzing six existing PPI databases including InAct, Human Protein Reference Database, Molecular interaction Database, Database of Interacting Proteins, Biological General Repository for Interaction Datasets and Biomolecular Interaction Network Database as described in Supplementary Table 10 [70].

### Network construction and its features

We first constructed an interaction network for the known CRC-related targets and predicted putative drug targets of TFS based on the data obtained from the Cytoscape plugin Bisogenet. The network was thereafter visualized with Cytoscape (Version 3.2.1) [71]. The topological property of each node in the interaction network was assessed by calculating six measures with a Cytoscape plugin CytoNCA, namely, ‘Degree centrality (DC)’, ‘Betweenness centrality (BC)’, ‘Closeness centrality (CC)’, ‘Eigenvector centrality (EC)’, ‘Network centrality (NC)’, and ‘Local average connec-tivity (LAC)’. The definitions and computational formulas of these parameters have been previously defined and represent the topological importance of a node in the network [54]. Larger the quantitative value, more important is the node in the network.

### Gene ontology and pathway enrichment analysis

Further, we performed gene ontology analysis using Omicsbean on the 468 putative targets of TFS to gain insights into their involvement in three different categories namely, biological processes (BP), molecular function (MF) and cell component (CC) [25]. We considered a P-value (have already been corrected through using Benjamini-Hochberg method) cut-off ≤ 0.05 and applied the hypergeometric test to identify the enriched GO terms. An overview of the gene ontology analysis with up to 15 significantly enriched terms in each of these three categories, respectively are shown in the chart (Supplementary Figure 2). The orders of terms in the same category were based on the *P*-values. The percentage of involved genes in a particular term is shown on the x-axis.

Then, we performed enrichment analysis of the 276 candidate targets of TFS using ClueGO, a Cytoscape plugin that visualizes non-redundant biological terms for large clusters of genes in a functionally grouped network [55]. The resultant candidates were divided into two categories, molecular functions/biological processes and the signaling pathway. The ClueGO network was created with kappa statistics and reflected the relationship between the terms based on the similarity of their associated genes.

### Cell culture and proliferation assays

Human CRC cells SW480, SW620, HT-29, HCT-116, DLD-1 and LS174T were purchased from Kunming Institute of Zoology, China. HCT-116 and DLD-1 cells (in RPMI 1640 medium), HT-29, SW480 and LS174T cells (in Dulbecco's Modified Eagle Medium), and SW620 cells (in Leibovitz's L-15 medium) were grown at 37°C in 5% CO_2_. All media were supplemented with 10% FBS and 1% penicillin/streptomycin. To determine cell proliferation, the cells (1 × 10^4^ cells/100 μL per well) were seeded into a 96-well flat-bottom plate. At 60% confluence, the cell plate was removed from the incubator, and the culture medium was removed by aspiration. Then, 100μL culture medium containing 10% FBS and 1% penicillin/streptomycin was added with or without 0.656~10.5 mg/ml TFS and the cell plate was placed into an IncuCyte ZOOM^®^ live cell imaging system set at 37°C for 30 min and scanned [72]. The detection parameters were set (objective: 4×; channel selection: phase contrast; scan type: standard; scan interval: 1 hr) and the plate was imaged accordingly until the end of the experiment at 48 h.

### The xenograft tumor transplant model

The animal studies were conducted according to the Guide for the Care and Use of Laboratory Animals of the National Institutes of Health according to the procedures approved by the Research Ethical Committee of Nanjing University of Chinese Medicine. The CRC cell lines, HT-29 and SW480 were cultured under normal conditions. A total of 80 (each xenograft model needed 40 mice) male BALB/c nude mice aged four- to five-week old and weighing 18–22 g were purchased from Vital River Laboratory Animal Technology Co., Ltd. (Beijing, China). The animals were maintained in a pathogen-free facility (23°C ± 2°C, 55% ± 5% humidity, 12 h light/12 h dark cycle) and injected subcutaneously with HT-29 and SW480 cells (2 × 10^6^ per mouse) into the abdomen and allowed 2 weeks to establish the tumors. Later, the xenografted mice were randomly divided into four groups of 10 mice each. The mice in the low-dose group (clinical dose) and high-dose group (2× clinical dose) were orally administered with 2.86 g/kg and 5.72 g/kg TFS once a day, respectively. Mice in the positive control group were intraperitoneally injected with 5 mg/kg oxaliplatin (clinical dose, once a week) and those in the model group were administered with the same volume of normal saline. The tumor size was measured by the vernier caliper twice a week and the tumor volume was calculated as 0.5 × L×W×H, where L is the tumor dimension at the longest point, W is the tumor dimension at the widest point, and H is the tumor dimension at the highest point. Relative tumor volumes were calculated as V_t_/V_0_ (V_0_ is the tumor volume when the treatment was initiated). Mice were weighed and normalized to their initial weights. Mice in each cohort were considered to be dead either when tumor volume increased to 1000 mm^3^ or when the mice died during treatment. The tumor weight was measured using an analytical balance after the mice were sacrificed through CO_2_ inhalation. All the tumors were bisected, with one part fixed in 10% formalin and paraffin-embedded for immunohistochemical and TUNEL staining and the other part snap-frozen and stored in liquid nitrogen for Western blot, ELISA and quantitative PCR analysis.

### Western blotting

Whole protein extracts were obtained by homogenizing frozen tumor samples in whole lysis buffer (10 mmol/L Tris-HCl, 250 mmol/L sodium chloride, 30 mmol/L sodium pyrophosphate, 50 mmol/L sodium fluoride, 0.5% Triton X-100, 10% glycerol, 1× proteinase inhibitor mixture, 1 mmol/L phenylmethylsulfonyl fluoride, 2 mmol/L iodoacetic acid, and 5 mmol/L ZnCl_2_). Protein concentrations were measured using a Thermo protein assay according to the manufacturer's instructions. For western blotting, 50 μg total protein from each sample was separated by sodium dodecyl sulfate-polyacrylamide gel electrophoresis and transferred onto polyvinylidene fluoride membranes using a wet transfer system (Bio-Rad, USA). The membranes were then blocked with 5% nonfat milk in TBST buffer [2.42 g/L Tris-HCl, 8 g/L NaCl, and 1 ml/L Tween 20 (pH 7.6)], incubated overnight at 4°C with primary antibodies suspended in TBST buffer, and then incubated with secondary antibody conjugated with horseradish peroxidase. Finally, the protein bands were detected by ChemiDoc^™^ XRS+ system (Bio-Rad, USA).

### Quantitative RT-PCR

The mRNA expression levels of genes were tested by SYBR green-based real-time quantitative PCR. Briefly, total cellular RNA from the lysates of transplanted tumors was extracted with chloroform solution after addition of Trizol reagent (Thermo Fisher Scientific, USA), precipitated with isopropanol solution and the RNA fraction dissolved in DEPC-H2O. An aliquot of 5 μg RNA was reverse-transcribed into cDNA with a HiScript II QRT SuperMix for qPCR (+gDNA wiper) kit (Vazyme, China). Quantitative RT-PCR was performed using a SYBR Green Master kit (Bio-Rad, USA) according to the manufacturer's instructions. The primer pairs are as shown in the Table 1. Gene expression levels of the samples were calculated relative to the control using the comparative CT method as follows: ΔΔCT = ΔCT sample – ΔCT control, fold change = 2^−ΔΔCT^. GAPDH expression was used as the internal control.

**Table 1:** PCR was performed using the follow primers

Gene name	PCR primers
GAPDH	5′-TGTGGGCATCAATGGATTTGG-3′
	5′-ACACCATGTATTCCGGGTCAAT-3′
CDK2	5′-CCAGGAGTTACTTCTATGCCTGA-3′
	5′-TTCATCCAGGG AGGTACAAC-3′
CDK4	5′-ATGGCTACCTCTCGATATGAGC-3′
	5′-CATTGGGGACTCTCACACTCT-3′
CCND1	5′-GCTGCGAAGTGGAAACCATC-3′
	5′-CCTCCTTCTGCACACATTTGAA-3′
CCND2	5′-CTGTCTCTGATCCGCAAGCAT-3′
	5′-GGTGGGTACATGGCAAACTTAAA-3′
BCL2	5′-TTCTTTGAGTTCGGTGGGGTC-3′
	5′-TGCATATTTGTTTGGGGCAGG-3′
BAX	5′-TCCACCAAGAAGCTGAGCGAG-3′
	5′-GTCCAGCCCATGATGGTTCT-3′
BCL2L1	5′-GAGCTGGTGGTTGACTTTCTC-3′
	5′-TCCATCTCCGATTCAGTCCCT-3′
XIAP	5′-AATAGTGCCACGCAGTCTACA-3′
	5′-CAGATGGCCTGTCTAAGGCAA-3′
VEGFA	5′-AGGGCAGAATCATCACGAAGT-3′
	5′-AGGGTCTCGATTGGATGGCA-3′
CDK6	5′-TCTTCATTCACACCGAGTAGTGC-3′
	5′-TGAGGTTAGAGCCATCTGGAAA-3′
FLT1	5′-TTTGCCTGAAATGGTGAGTAAGG-3′
	5′-TGGTTTGCTTGAGCTGTGTTC-3′
KDR	5′-GTGATCGGAAATGACACTGGAG-3′
	5′-CATGTTGGTCACTAACAGAAGCA-3′
MMP9	5′-AGACCTGGGCAGATTCCAAAC-3′
	5′-CGGCAAGTCTTCCGAGTAGT-3′
HIF1A	5′-ATCCATGTGACCATGAGGAAATG-3′
	5′-TCGGCTAGTTAGGGTACACTTC-3′
PTGS2	5′-TA AGTGCGATTGTACCCGGAC-3′
	5′-TTTGTAGCCATAGTCAGCATTGT-3′
NOS2	5′-TTCAGTATCACAACCTCAGCAAG-3′
	5′-TGGACCTGCAAGTTAAAATCCC-3′
MYC	5′-GTCAAGAGGCGAACAC ACAAC-3′
	5′-TTGGACGGACAGGATGTATGC-3′
BIRC5	5′-AGGACCACCGCATCTCTACAT-3′
	5′-AAGTCTGGCTCGTTCTCAGTG-3′
TLR4	5′-AGACCTGTCCCTGAACCCTAT -3′
	5′-CGATGGACTTCTA AACCAGCCA -3′
RELA	5′-AACAGAGAGGATTTCGTTTCCG -3′
	5′-TTTGACCTGAGGGTAAGACTTCT -3′

### ELISA

The levels of inflammation -related factors: IL-1α, IL-1β, IL-2, IL-6, IL-10 and TNF-α in the whole protein extracts from the tumor samples were detected with a commercial ELISA kit (R&D Systems, USA). The absorbances of all samples were measured by a microplate reader at 490 nm.

### IHC Staining

Serial sections (4 μm) were cut from formalin-fixed, paraffin-embedded CRC xenograft tissue samples for IHC staining. Further, after retrieving the antigen using citrate buffer (0.01 ml, pH 6.0), the sections were washed three times with PBS and incubated in 10% normal goat serum to block non-specific background staining. The sections were then incubated overnight with rabbit anti-human antibodies (Abcam, USA) at 4°C. Further, after washing thrice with PBS, the sections were incubated with horseradish peroxidase-labeled anti-rabbit IgG antibody (Bioworld, USA) for 30 min at room temperature, washed with PBS and developed using diaminobenzidine. Three middle-power microscopic fields (×200) were randomly selected for each section to determine the positive staining intensities by IPP software (Image-Pro Plus 6.0, Media, Cybernetics) and the integrated optical density (IOD) was calculated. The expression levels of the proteins were presented as the average IOD of the 3 fields.

### Cell cycle assay

HT-29 cells were seeded in 6-well plates at concentrations of 2 × 10^5^ per well for 24 h and then treated with 1.31, 2.63 or 5.25 mg/ml TFS for another 24 h. The negative control was culture medium only. The cells were then collected, centrifuged, and fixed overnight with 70% ethanol at 4°C. Finally, the cells were stained by propidium iodide (Keygen Biotech, China) solution (1 mg/ml PI in100 μl per well) at room temperature for 30 min in darkness. Cell cycle analysis was performed by FACS using appropriate controls (BD Accuri C6, USA).

### Apoptosis assays

Apoptosis was detected by double staining with annexinV and PI. HT-29 cells were placed in 6-well culture plates at concentrations of 2 × 10^5^ per well. After 24 h of incubation, the cells were exposed to 1.31, 2.63 or 5.25 mg/ml TFS and cultured for 24 h. The wells treated with culture medium without TFS were used as the negative control. The cells were then harvested by centrifugation and their cell concentration was adjusted to 10^9^/L. Subsequently, the cells were stained with annexinV-FITC/PI apoptosis detection kit (KeyGEN BioTECH, China) and analyzed by FACS (BD Accuri C6, USA) according to the manufacturer's instructions.

TUNEL assay was performed to determine apoptosis in the tissue samples. Tissue sections (4 μm) were tested using an *in situ* cell death detection kit (Roche, Mannheim, Germany) according to the procedures described by the manufacturer. Under a high-power microscope, 3 randomly chosen fields (×200) without any necrotic areas were observed and the average percentage of positive cells in the 3 fields for each section was determined.

### Tube formation assay

Conditional medium was collected from HT-29 cells pretreated with/without TFS for 24 h. Matrigel (Becton Dickinson, Bedford, MA, Cat# 356231 ) was thawed, mixed with an equal volume of complete medium containing 2 × 10^4^ of HUVEC and treated with conditioned media. Tube formation was quantified by counting the number of branch points that formed from complete branching per field of view. Five fields of view were determined for each treatment in replicate.

### Statistical analysis

All data were expressed as percentage and mean with standard deviations ( ± SD). Statistical analysis was performed using Student's *t*-test and one-way ANOVA by GraphPad Prism 5 for Windows. A *p* < 0.05 was considered as statistically significant.

## SUPPLEMENTARY MATERIALS FIGURES AND TABLES








































