# Supplementary material for: Tianfoshen oral liquid: a CFDA approved clinical traditional Chinese medicine, normalizes major cellular pathways disordered during colorectal carcinogenesis
Source: Oncotarget. 2017 Jan 16;8(9):14549–69. doi: 10.18632/oncotarget.14675 (PMC5362425; doi:10.18632/oncotarget.14675)
Supplement: Supplementary file 1 [file oncotarget-08-14549-s001.pdf]

Tianfoshen oral liquid: a CFDA approved clinical traditional Chinese medicine, normalizes major cellular pathways disordered during colorectal carcinogenesis

Supplementary Materials

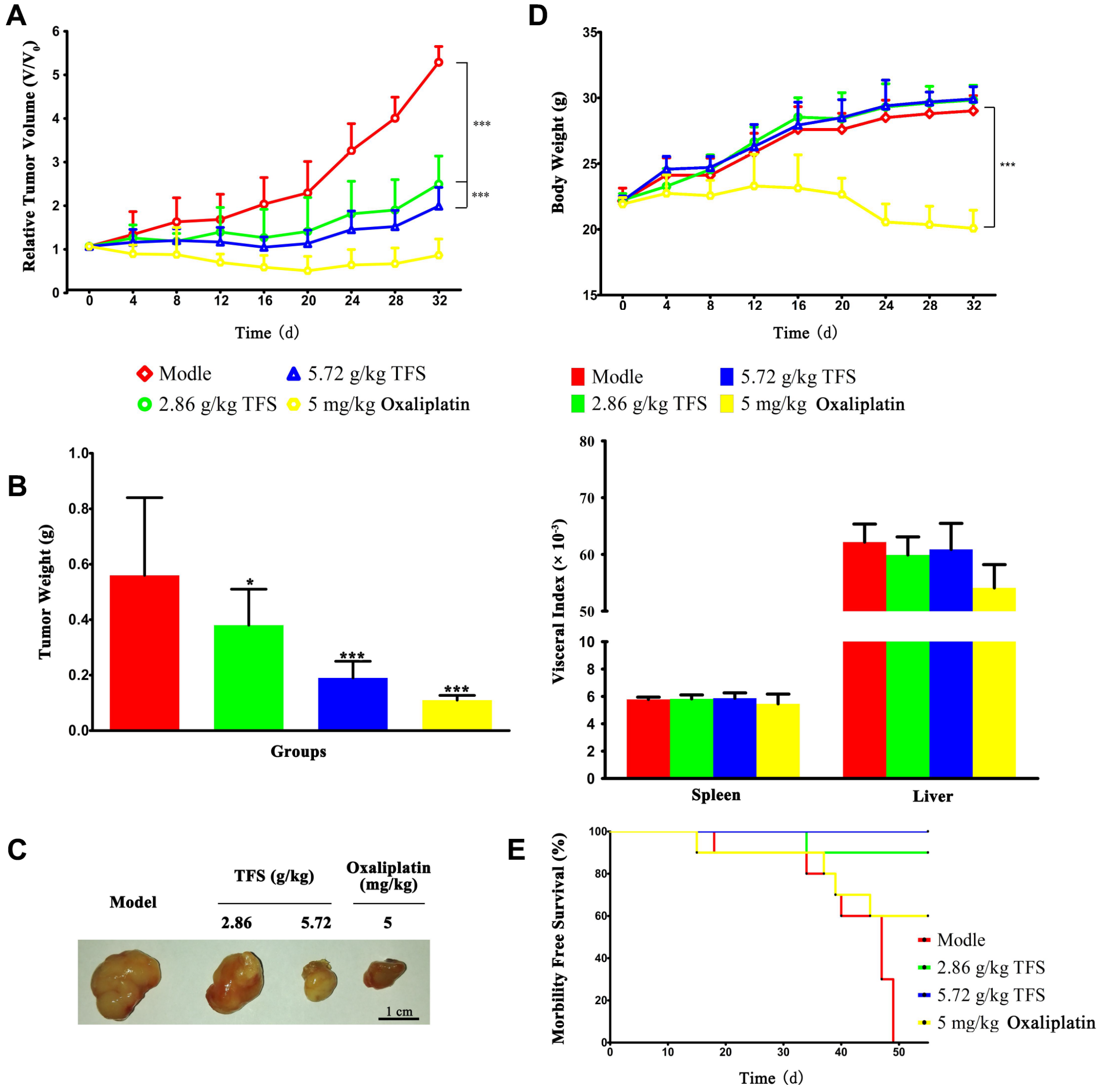

Supplementary Figure 1: Effect of TFS on SW480 exnograft tumor. (**A**) Tumor volume changes of mice treated with TFS (2.86 g/kg and 5.72 g/kg), oxaliplatin (5 mg/kg) and normal saline (model), respectively. Data are presented as mean ± SD (n = 10). ***$P < 0.001$ (versus model). (**B**) Weight of tumor collected from different treatment groups of mice on day 32. Data are presented as mean ± SD (n = 10). ***$P < 0.001$ (versus model). (**C**) Photographs of typical tumor blocks collected from different treatment groups of mice on day 32. (**D**) Body weight (above) and visceral index (below) changes of mice in different treatment groups on day 32. Data are presented as mean ± SD (n = 10). ***$P < 0.001$ (versus model). (**E**) Survival rates of mice in different treatment groups within 55 d.

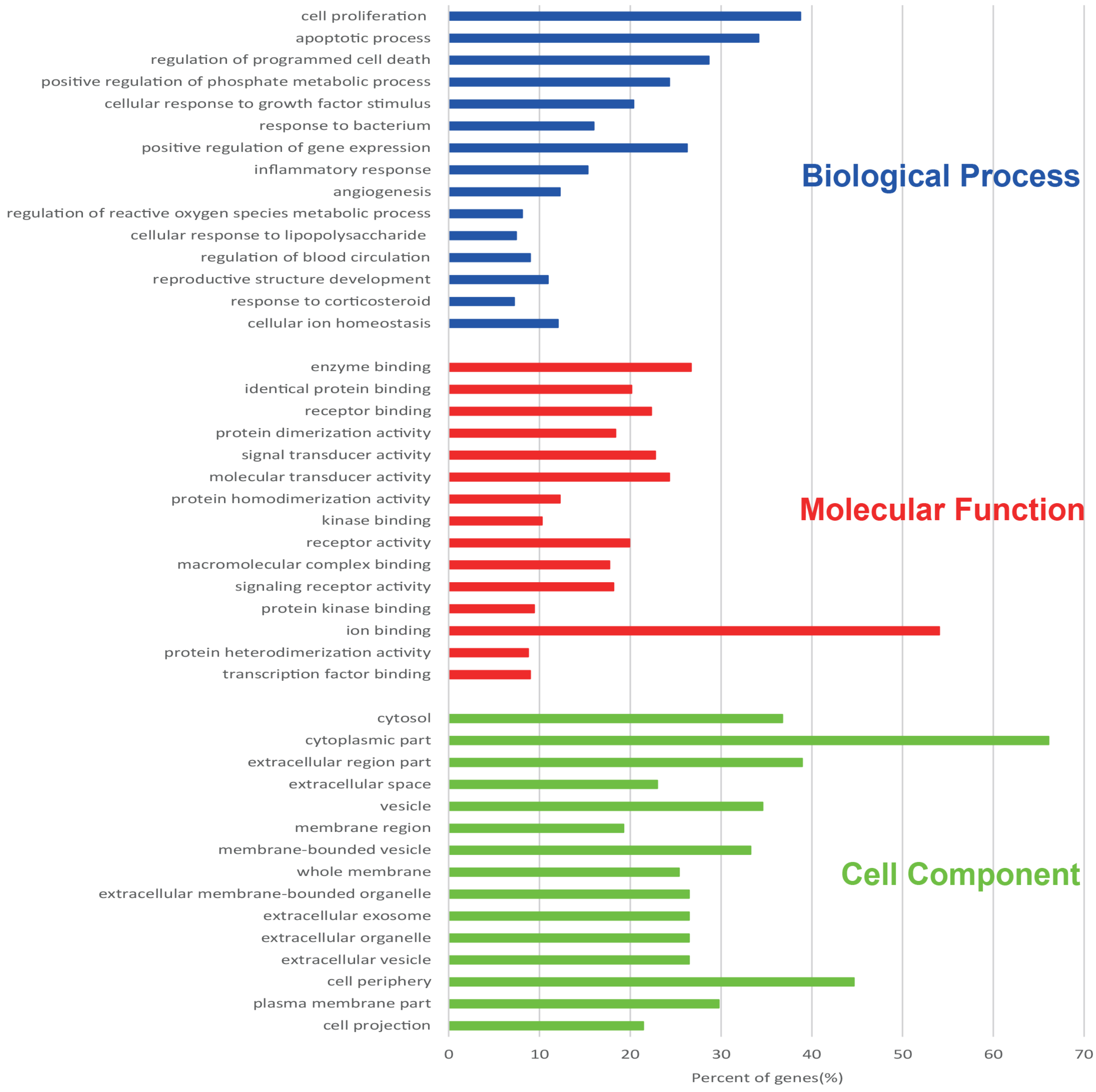

Supplementary Figure 2: Gene ontology analysis of the putative targets of TFS. 468 putative targets of TFS was performed in Omicsbean to gain more insights into their involvement in various Biological Processes (blue section), Molecular Function (red section) and Cell Component (green section). We considered a P-value (have already been corrected through using Benjamini-Hochberg method) cut-off of ≤ 0.05 as significant and applied hypergeometric test to identify enriched GO terms. Following chart shows an overview of the gene ontology analysis with up to 15 significantly enriched terms in each of these three categories, respectively. Terms of same category are ordered by *P*-values, above terms are more significant. Information of the percentage of involved genes in a term are shown in x-axis.

Supplementary Table 1: Candidate compounds of each ingredient in TFS. See_Supplementary_Table 1

Supplementary Table 2: Putative targets of TFS. See_Supplementary_Table 2

Supplementary Table 3: The candidate compounds of each herb in TFS and their potential targets. See_Supplementary_Table 3

Supplementary Table 4: Putative targets overlaps among each herb and other seven ones of TFS. See_Supplementary_Table 4

Supplementary Table 5: The parameter of nodes in compound-putative target network. See_Supplementary_Table 5

Supplementary Table 6: Known CRC-related targets. See_Supplementary_Table 6

Supplementary Table 7: TFS shared 78 putative targets with known anti-CRC drugs. See_Supplementary_Table 7

Supplementary Table 8: Topological feature values of candidate targets for TFS against CRC. See_Supplementary_Table 8

Supplementary Table 9: Compositive compounds of each ingredient in TFS. See_Supplementary_Table 9

Supplementary Table 10: Detailed information on six existing protein-protein interaction databases

Source_name	Website	Release number/ Last updated
InAct	http://www.ebi.ac.uk/intact/	Version 4.2.3.2
Human Protein Reference Database (HPRD)	http://www.hprd.org/	Release 9
Molecular INTeraction Database (MINT)	http://mint.bio.uniroma2.it/mint/download.do	Aug-2011
Database of Interacting Proteins (DIP)	http://dip.doe-mbi.ucla.edu/dip/	Jan-2010
biomolecular interaction network database (BIND)	http://www.bind.ca	Version 2.0
Biological General Repository for Interaction Datasets (BIOGRID)	http://thebiogrid.org/	Version 3.4.132

Supplementary Table 11: Molecular functions/biological processes results analysised by GluGO

GOTerm	Term *P* Value Corrected with Bonferroni step down
Cyclin-dependent protein serine/threonine kinase activity	1.14E-8
Apoptosis	1.27E-7
Vascular endothelial growth factor-activated receptor activity	4.29E-7
Cytokine secretion	4.64E-6
Protein localization to plasma membrane	4.29E-5
Mitotic DNA damage checkpoint	5.98E-5
Transmembrane receptor protein tyrosine kinase activity	7.23E-5
Cytochrome c release from mitochondria	1.27E-4
Apoptotic DNA fragmentation	3.51E-4
Endothelial cell migration	4.10E-4
Cell-matrix adhesion	4.10E-4
Icosanoid biosynthetic process	4.41E-4
Epithelial to mesenchymal transition	4.47E-4
Unsaturated fatty acid biosynthetic process	8.17E-4
Cytokine biosynthetic process	0.001
Deoxyribonuclease activity	0.004
Lipid biosynthetic process	0.006
Collagen biosynthetic process	0.006
Histone deacetylation	0.009
Leukotriene metabolic process	0.011

Supplementary Table 12: Pathway results analysised by GluGO

GOTerm	Term *P* Value Corrected with Bonferroni step down
MAPK signaling pathway	1.74E-16
Cell cycle	1.46E-11
IL-2 Signaling Pathway	5.19E-10
EGF/EGFR Signaling Pathway	8.80E-9
Apoptosis	3.61E-8
Cytokines and Inflammatory Response	9.89E-8
Integrated Cancer pathway	1.22E-7
DNA damage response (only ATM dependent)	1.25E-7
angiogenesis overview	9.75E-6
Toll-like receptor signaling pathway	1.04E-5
Wnt Signaling Pathway	1.40E-5
TP53 network	2.79E-5
Estrogen signaling pathway	4.23E-5
Oxidative Stress	5.76E-5
Matrix Metalloproteinases	6.38E-5
Senescence and Autophagy	7.76E-5
Actin Cytoskeleton	1.06E-4
Notch Signaling Pathway	1.26E-4
G1 to S cell cycle control	4.07E-4
miRNA regulation of DNA Damage Response	4.26E-4

Supplementary Video 1: Representative time-lapse movies of DLD-1 treated with-out TFS for 48 h (100×). See Supplementary_Video_1

Supplementary Video 2: Representative time-lapse movies of DLD-1 treated with 2.63 mg/ml TFS for 48 h (100×). See Supplementary_Video_2

Supplementary Video 3: Representative time-lapse movies of HT-29 treated with-out TFS for 48 h (100×). See Supplementary_Video_3

Supplementary Video 4: Representative time-lapse movies of HT-29 treated with 2.63 mg/ml TFS for 48 h (100×). See Supplementary_Video_4

Supplementary Video 5: Representative time-lapse movies of LS174T treated with-out TFS for 48 h (100×). See Supplementary_Video_5

Supplementary Video 6: Representative time-lapse movies of LS174T treated with 2.63 mg/ml TFS for 48 h (100×). See Supplementary_Video_6

Supplementary Video 7: Representative time-lapse movies of NCM460 treated without TFS for 48 h (100×). See Supplementary_Video_7

Supplementary Video 8: Representative time-lapse movies of NCM460 treated with 10.5 mg/ml TFS for 48 h (100×). See Supplementary_Video_8

Supplementary Video 9: Representative time-lapse movies of SW480 treated without TFS for 48 h (100×). See Supplementary_Video_9

Supplementary Video 10: Representative time-lapse movies of SW480 treated with 2.63 mg/ml TFS for 48 h (100×). See Supplementary_Video_10
